# Personalized diet study of dietary advanced glycation end products (AGEs) and fatty acid desaturase 2 (FADS_2_) genotypes in obesity

**DOI:** 10.1038/s41598-021-99077-3

**Published:** 2021-10-05

**Authors:** Mahsa Mahmoudinezhad, Mahdieh Abbasalizad Farhangi, Houman Kahroba, Parvin Dehghan

**Affiliations:** 1 Department of Community Nutrition, Faculty of Nutrition, Tabriz University of Medical Sciences, Attar-Neishabouri Ave, Golgasht St, 5165665931 Tabriz, Iran; 2 Molecular Medicine Research Center, Biomedicine Institute, Tabriz University of Medical Sciences, Tabriz, Iran; 3 Department of Biochemistry and Nutrition, Faculty of Nutrition, Tabriz University of Medical Sciences, Attar-Neishabouri Ave, Golgasht St, 5165665931 Tabriz, Iran

**Keywords:** Biochemistry, Biotechnology, Genetics, Molecular biology, Biomarkers, Diseases, Endocrinology, Risk factors

## Abstract

Obesity prevalence have tripled in the past decades. It is logical to consider new approaches to halt its prevalence. In this concept, considering the effect of interaction between fatty acid desaturase 2 (FADS_2_) gene variants and dietary advanced glycation end products (AGEs) on obesity-related characteristics seems to be challenging. The present cross-sectional study conducted among 347 obese individuals. A validated semi-quantitative 147-item food frequency questionnaire (FFQ) was used to estimate dietary intakes and American multiethnic database was used to calculate AGEs content of food items which were not available in Iranian Food Composition Table (FCT). FADS_2_ gene variants were determined according to Polymerase chain reaction-restriction fragment length polymorphism (PCR-RFLP). Analysis of covariance (ANCOVA) was used to evaluate the modifier effect of FADS_2_ gene-dietary AGEs on biochemical values. Based on our findings, no significant differences was reported in term of biochemical variables between AGEs tertiles. In contrast, percent of macronutrients (carbohydrate, protein and fat) of total calorie intake, amount of daily intake of fiber and meat groups showed a significant differences among AGEs tertiles. Furthermore, statistical assays clarified the modifier effects of FADS_2_ gene-AGEs on weight (P_interaction_ = 0.04), fat mass (P_interaction_ = 0.03), waist circumference (P_interaction_ = 0.008) and cholesterol (P_interaction_ = 0.04) level. Accordingly, higher consumption of protein or fat based foods constitute high amount of AGEs and heterozygote genotype for FADS_2_ tended to show lower level of AGEs content. These findings address further investigation to develop new approaches for nutritional interventions.

## Introduction

**Figure1:**
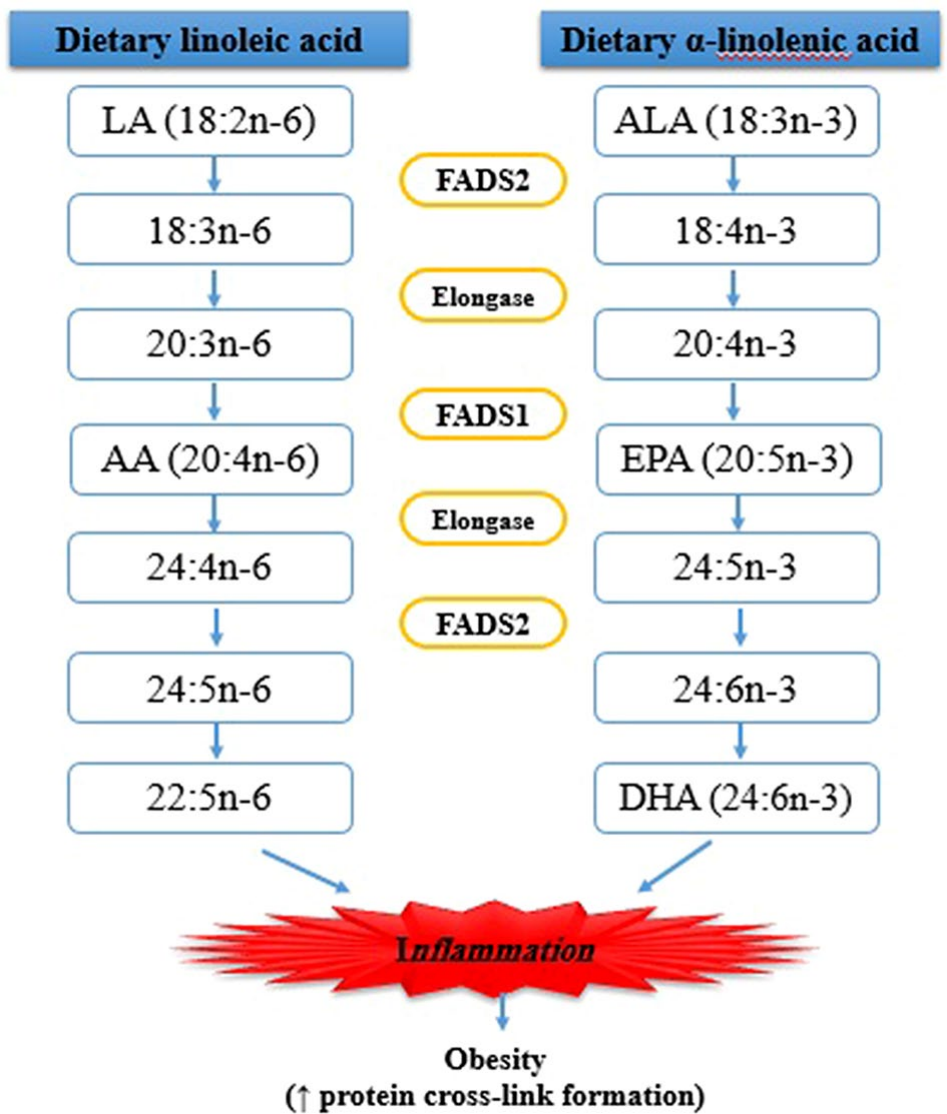
Mechanisms of AGEs involved in obesity progression.

Obesity represent world health concern and it has been known as “globesity” according to dramatic increasing prevalence during the last two decades and Iran has not been exception with 22.3% prevalence^1,2^. In addition, world Obesity Federation have introduced obesity as a chronic progressive disease not just a risk factor for non-communicable diseases such as diabetes mellitus, hypertension, fatty liver disease, stroke, myocardial infarction and cancers in industrialized countries^3,4^. Obesity is a multifaceted disease that affects different aspects of life including: unemployment, social disadvantages and reduced socio- economic productivity in long term. With respect to its important health repercussions, world health organization (WHO) considered obesity as a priority of “Global Action Plan for the Prevention and Control of NCD; 2013–2020” to attenuate its increasing trend and detrimental effects^3^. According to WHO definition, overweight is defined as a body mass index (BMI) ≥ 25 and obesity as BMI ≥ 30 in adults. Based on above introduction, lifestyle modification is needed to overcome its prevalence. However, considering only life style modification such as energy restriction or increased physical activity has not been shown to be effective. Evidence show that obesity is a result of metabolic, hormonal and genetic interactions. There are some genes affecting body’s response to environment and understanding inter-personal dynamics and keeping the role of diet alongside is a useful approach for nutritional interventions. Prior studies revealed the role of fatty acid desaturase 2 (FADS_2_) gene polymorphism in fat metabolism which affects cardio-metabolic markers^5^. FADS_2_ gene encode delta 6-desaturase enzyme, involved in the desaturation steps of dietary linoleic acid and α-linolenic acid to produce long chain poly unsaturated fatty acids (LC-PUFA)^6–8^. Several studies point out that FADS_2_ gene polymorphism may interact with dietary intakes which results in metabolic factors modification^5,9^ (Fig. 1). On the other hand, lifestyle changes in Iranian population alongside with industrialization have led to having short time for food preparation and a great desire in processed foods with high fat and sugar content; this dietary transition have led to change in dietary patterns and preferences which is mainly composed of high carbohydrate and fat contributing to increased production of advanced glycation end products (AGEs). AGEs, a heterogeneous group of molecules, are formed through irreversible covalent bonds’ formation between reduced sugar and free amino group of protein, nucleic acid and lipids^10,11^. These glycotoxins, are produced in both endogenous and exogenous pathways. The oxidative and non-oxidative chemical reactions ultimate to the production of endogenous AGEs^12^. Nonenzymatic reactions between reactive sugars and proteins (e.g.Maillard reaction) contribute to AGEs formation^13^. Likewise, the Maillard reaction, leading to food browning, is known to increase AGEs formation and this phenomenon in proteins and reducing sugars are accelerated depending on temperature, time of food preparation, pH and size of the sugars^14–16^. Furthermore, the role of foods as well as smoking and air pollution as exogenous sources of AGEs have been illustrated^12^. The endogenous or exogenous origins of AGEs and whether they are in free or protein bound form, determine the features of every AGEs and affect the ability for cross-link proteins.

**Figure 2:**
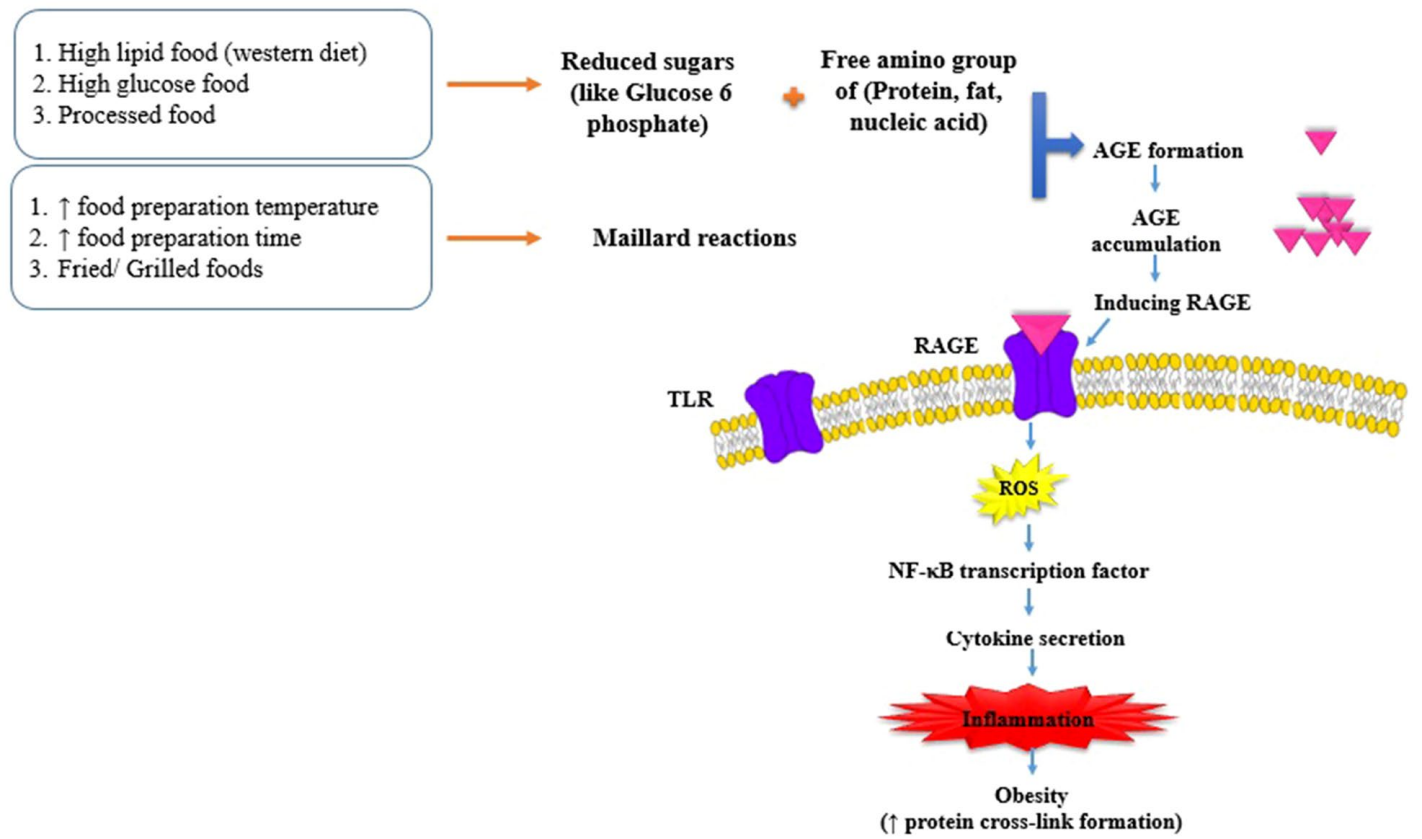
The role of FADS2 gene in polyunsaturated fatty acids promoting obesity.

The majority of reports known to date, point to the effect of AGEs in metabolic diseases^15^. Tessier et al. evaluated the amount of Carboxymethyl-lysine (CML) levels, as the most known AGEs in the body tissues of mice in oral exposure to dietary CML; they also reported a higher accumulation of CML in the kidney, intestine and lungs. This indicates that significant amount of absorbed AGEs products are likely to be retained in the body and lower amounts are eliminated from urine. Although Tessier et al. reported lower amount of CML in the adipose tissue of animals despite chronic oral exposure to CML^17^, however, in another study conducted by Gaens KHJ et al., the CML amount in the adipose tissue of obese individuals was meaningful^18^. AGEs are produced through physiological processes during normal metabolic state, however, exposure to excessive amounts of AGEs, particularly when they are not detoxified, may contribute to pathological disorders and stimulate inflammatory response and oxidative stress commonly associated with obesity^19,20^. AGEs binding to their receptors (RAGE) will facilitate the production of reactive oxygen species (ROS) and inflammatory cascades including RAGE/ Toll-like receptors 4 (TLR4) and nuclear factor κB (NF-κB) transcription factor production^15,20^ (Fig. 2). Prior studies suggest that two neurohormones which are regulating food behaviors including agouti related peptide (Ag-RP) and α-melanocyte stimulating hormone (α-MSH), may be affected through inflammatory status promoted by AGEs too^21,22^. In another glance, aldehydes content of AGEs processing may contribute to increased level of oxidative stress and obesity consequently. In this pathway, glucose to sorbitol conversion reduces the precursor that are needed for glutathione (GSH) production; which is the most likely mechanism to increased polyol pathway flux, contributing to obesity^23^. Since dietary AGEs increase the risk of obesity, lower intake of dietary AGEs may be beneficial to human health independent of energy restriction; even tough, AGEs have been introduced as a novel biomarker of obesity^14^. Accordingly, this study with a new gene-nutrient approach, can provide a better perspective of dietary interventions and it is of interest that whether FADS_2_ gene polymorphism and dietary AGEs content might affect obesity-related risk-traits together which has not been assessed before.

## Method and materials

### Study population

The present cross-sectional study is a combination of two projects with 188 and 158 obese participants conducted in Tabriz, Iran. All 347 subjects were in the age range of 20 to 50 years old and met the inclusion criteria, BMI (30–40) kg/m^2^. Individuals who met the following criteria were excluded from the study: past medical history of cardiovascular diseases, diabetes mellitus, kidney disorders, pregnancy, lactation and taking any medication that affect weight status. The written informed consent was completed by all participants and the study protocol was approved by the ethics committee of Tabriz University of Medical Science (Identifier: IR.TBZMED.REC.1399.062 and IR.TBZMED.REC.1400.454). Also, we confirm that all methods were performed in accordance with the relevant guidelines and regulations.

### Anthropometric and biochemical assessments

A questionnaire containing demographic characteristics and socio-economic status (SES) had been prepared and was completed by face to face interviewing. In addition, the International Physical Activity Questionnaire (IPAQ) was used to evaluate the physical activity status^24^. The anthropometric assays including: weight, height, BMI, waist circumference (WC) and hip circumference (HC), were measured by a trained one to record accurate measurements. Weight measurement was conducted using Seca scale (Seca, Germany) with light clothing (near to 100 g). Height assessment was done by stadiometer with standing on heels (near to 0.5 cm). A non-stretchable tape was used to estimate the WC by keeping it around the middle, above the umbilicus (near to 0.1 cm)^25^ and HC, the largest circumference of buttocks. BMI was calculated as body weight in kg divided to height in meter squared. Bioelectrical impedance analysis (BIA) was used to estimate body composition. Moreover, blood pressure was measured as a vital sign in a proper condition, while subjects were sitting on a chair with supporting the arm at the heart level quietly for 5 min before the test. To evaluate biochemical values, 10 ml fasting blood was obtained from participants. Bloods were centrifuged at 4500 rpm at 4 °C for 10 min to isolate serum and plasma. Glucose and lipid profile markers including: triglyceride (TG), total cholesterol (TC), high-density lipoprotein cholesterol (HDL-C) concentration were measured using a commercial kit (Pars Azmoon, Tehran, Iran). Serum low-density lipoprotein cholesterol (LDL-C) was calculated using Friedewald’s equation^26^. Moreover, homeostasis model assessment-insulin resistance index (HOMA-IR) and quantitative insulin sensitivity check index (QUICKI) were calculated as insulin resistance and sensitivity indices respectively^27,28^. Commercially available enzyme-linked immunosorbent assay kits (Bioassay Technology Laboratory, China) were used to evaluate plasma Ag-RP and α-MSH concentrations according to manufacturer’s protocol. The sensitivity of this assay for Ag-RP and α-MSH was 5.07 ng/L and 1.03 pg/ml respectively.

### Dietary assessment

Dietary assessment was done to estimate the frequency and amount of each 147 food items consumed over the past year, on a daily, weekly, monthly or yearly basis using a validated semi-quantitative food frequency questionnaire (FFQ) by a trained dietitian^29,30^. All recorded responses were converted to grams per day using handbook of household measurements^31^. Energy and nutrient consumption was analyzed by Nutritionist IV software. We calculated the AGEs content of foods (per day) considering AGEs content of each food item in kilo-unit/gram. Regarding to Iranian Food Composition Table (FCT) which does not contain AGEs content of foods, we used the data of AGEs for 549 routine food items provided for Northeastern American multiethnic population^16,32^. The first published report about the AGEs content of food was brief and comprised from only several food items^32^, while the new database is more comprehensive and includes 549 food items of different food groups (e.g. all of fats, fat liquids, all meats and meat substitutes, carbohydrates, fruits, fruit juices, liquids and combination foods and solid condiments, soups, liquid condiments, and miscellaneous liquids). The highest amount of AGEs in food items belong to meat and meat products such as beef and cheeses and then poultry, pork, fish, and eggs; although there were several differences between the food items consumed by Iranians and Americans, but, the N4 software is completely adopted to Iranian food habits and so, it has been considered to minimize the possible difference between estimated and actual amounts of consumed AGEs in diet. The table provided by Uribarri et al.^16^ for AGEs contents of food is very comprehensive comprising from more than 549 food items with different preparation methods that considers even the duration of cooking and temperature that makes a reliable adjustment for possible difference in Iranian food preparation habits. According to dietary differences among population, some food items such as types of Iranian breads, were not available in the table of American AGEs content of foods, so we replaced the most similar food items and adopted them according to the Iranian food staple. Also, some of food items that were not available in our FFQ were considered as missing items.

### Genotyping

**Figure 3:**
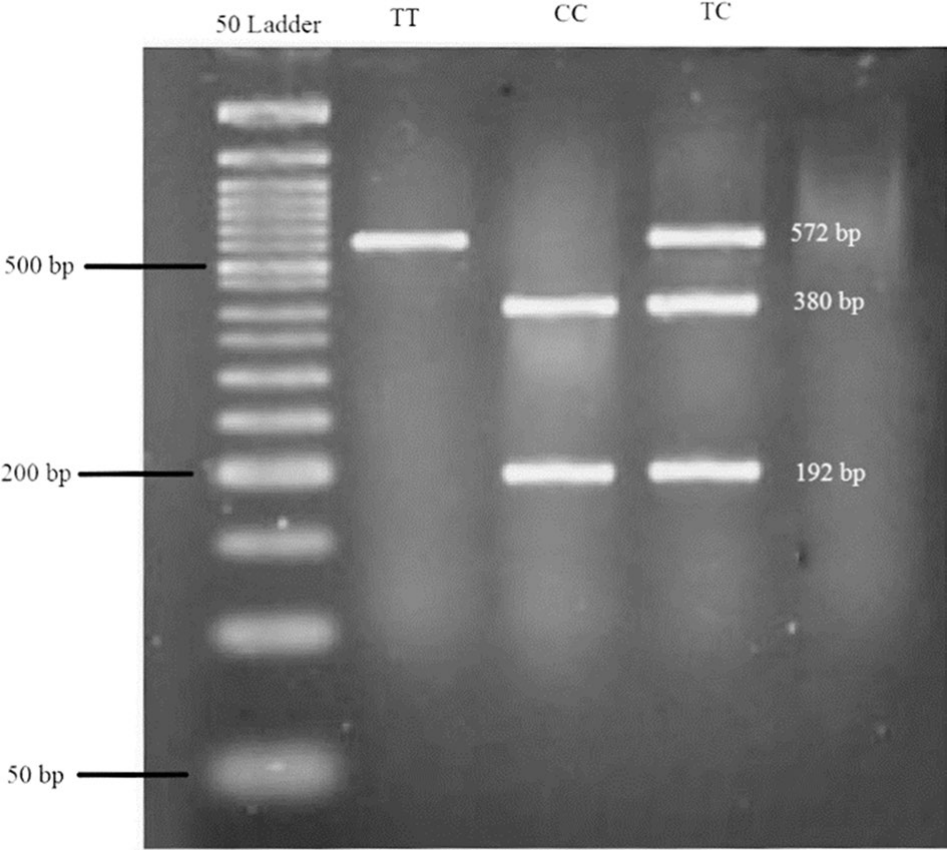
Electrophoretic gels and blots for FADS2 gene.

Single nucleotide polymorphism (SNP) was chosen from studies that were related to obesity^9,33^. Genomic DNA was extracted using whole blood with chloroform technique. All DNAs genotyped for rs174583, located in chromosome 11 of FADS_2_, using polymerase chain reaction-restricted length polymorphism (PCR–RFLP) method (Fig. 3). The primers with following sequence: forward, 5′ AGGAAGCAGACCACAGAGTC 3′; reverse, 5′ TCCTTCGTCTGGTGTCTCAG 3′ were used for PCR. PCR reactions contained: 5 μl Master Mix (Ampliqon; Denmark), 2 μl extracted DNA, 1 μl primers and 2 μl distilled water. The DNA thermocycler (BIO RAD T100 Thermal Cycler), that was used to PCR cycling, was set-out according to following command: 95 °C for 10 min of denaturation, amplification consisted of 35 cycles at 94 °C, annealing at 60 °C for 20 s and 50 s of extension at 74 °C and final extension occurred at 74 °C for 10 min. At the rest, TauI (cat. Num ER1652, USA) was used to digest restriction sites on the DNA. Fragments containing three possible genotypes of the FADS2 rs2239670 were detected: uncut homozygous TT (572 bp), cut heterozygous TC (192, 380 and 572 bp) and cut homozygous CC (192, 380 bp) (Supplementary Fig. 1). Finally, green stained gel of electrophoresis in a Gel-Doc-system (U.V.P Company, Cambridge, UK) was used to see digested PCR products.

### Statistical assay

Statistical analysis were performed using SPSS software (USA, version 25). Sample size of the present study was calculated according to the study of Hu et al.^34^ using G-power software, considering α = 0.05, β = 0.20, and power 80% and the correlation equation of r = 0.25. Accordingly, the calculated sample size was estimated as 300 individuals. Considering the drop-out rate of 15%, a total of 345 sample was needed to accomplish the study. The sample size was then divided into tertiles which was made according to the power of 80%. Normal distribution of variables were checked due to mean, standard deviation (SD), skewness and kurtosis and all reported as mean ± SD for normal distributed and median (min, max) for non-normal parameters respectively. Categorical values were reported as number and frequency (%) using χ^2^ test. The comparison of parameters across different AGEs tertiles was performed using One-way ANOVA and Kruskal–wallis tests. ANCOVA multivariate interaction model was also used to investigate the interaction between the FADS_2_ rs174583 polymorphism and dietary AGEs content in terms of metabolic risk factors and their interaction are presented as illustrative graphs. P value ≤ 0.05 was considered to be statistically significant.

## Results

**Figure 4:**
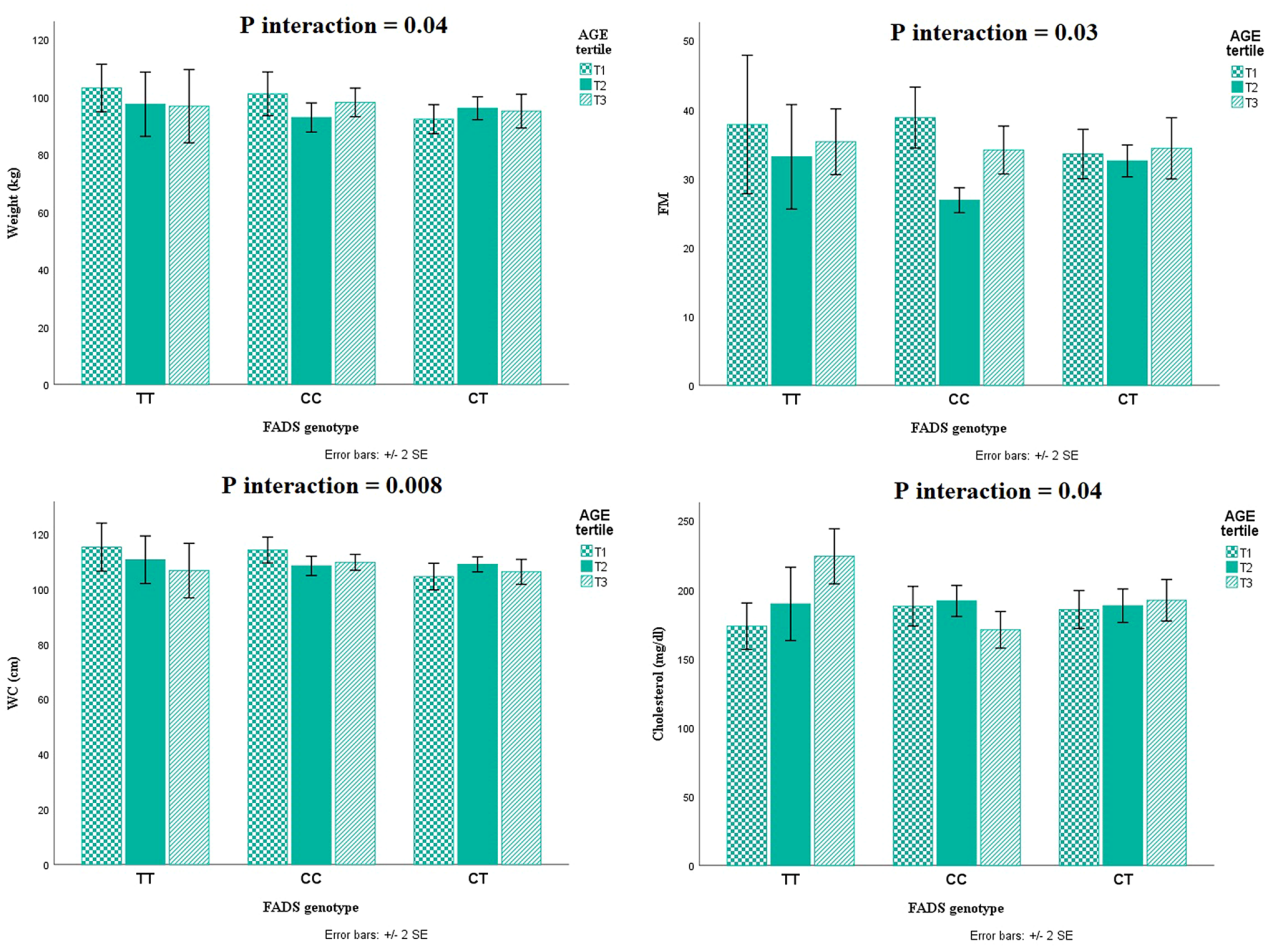
Interactions between FADS2 genotypes and different dietary AGEs tertiles to modify metabolic factors.

The mean dietary intake of AGEs in men and women was 3.25 ± 1.47 and 3.11 ± 1.49 kilo unit/gram respectively. Basic characteristics of all 347 study participants across dietary AGEs tertiles are provide in Table 1. Accordingly, individuals with higher age had lower dietary AGEs consumption and were more cautious about their dietary preferences but younger adults were eager to follow western diet with high fat and carbohydrate content. In addition, statistical analysis revealed a significant difference between weight, fat mass (FM), systolic blood pressure (SBP) and diastolic blood pressure (DBP) across different AGEs tertiles (P < 0.05). Also, we stratified the results according to gender and the results are provided in Supplementary Table 1. According to the results of gender-stratified comparison of parameters, there were no significant difference in separate analysis between biochemical parameters across different AGEs tertiles except for FM and SBP in men (P < 0.05). Table 2 shows the status of biochemical values and pro-inflammatory cytokines among AGEs tertiles; no significant difference had been reported. Table 3 represents the percent of daily macronutrients [carbohydrate (P < 0.001), protein (P = 0.005) and fat (P < 0.001)], fiber and meat groups. Based on recorded results, it shows that people with high consumption of dietary fat have increased level of dietary AGEs. The difference between AGEs tertile about carbohydrate, protein, fat, fiber and meat groups are statistically significant (P < 0.05). Furthermore, statistical analysis illustrated that individuals with TT genotypes had higher adherence to western diet with high AGEs content (Table 4), although this difference was not significant between groups. Figure 4 indicates the significant interactions between FADS_2_ gene variants and AGEs tertiles in terms of body weight (P_interaction_ = 0.04), FM (P_interaction_ = 0.03), WC (P_interaction_ = 0.008) and cholesterol (P_interaction_ = 0.04) even after adjusting for potential confounders. In this regard, it is visible that TT carriers are more prone to have higher WC even despite low dietary AGEs consumption. Similarly, TT carriers had experienced higher level of blood cholesterol by increased dietary AGEs amount.

**Table 1:** Comparison of demographic characteristics and anthropometric values in AGEs tertiles.

Variables	AGEs (kilo unit/gram)			P*
	Tertile 1 (< 2.45)	Tertile 2 (2.46–3.58)	Tertile 3 (> 3.59)	
**Age (year)**	42.49 ± 8.66	39.94 ± 8.70	39.08 ± 9.44	**0.01**
**Sex**				0.18
Male, n (%)	56 (29.2)	74 (38.5)	62 (32.3)	
Female, n (%)	52 (36.1)	42 (29.2)	50 (34.7)	
**SES, n (%)**				
Low	3 (60)	1 (20)	1 (20)	0.42
Middle	28 (28.3)	35 (35.4)	36 (36.4)	
High	20 (24.1)	36 (43.4)	27 (32.5)	
**Physical activity, n (%)**				0.52
Low	26 (28.9)	35 (38.9)	29 (32.2)	
Moderate	12 (23.1)	17 (32.7)	23 (44.2)	
High	13 (28.3)	20 (43.5)	13 (28.3)	
**Weight (kg)**	91.88 ± 8.66	92.05 ± 14.36	92.20 ± 14.21	0.98
**BMI (kg/m**^**2**^**)**	33.10 ± 5.36	32.31 ± 4.36	32.62 ± 4.79	0.47
**FM**	36.49 ± 9.05	30.94 ± 7.17	34.87 ± 10.31	**0.002**
**FFM**	60.66 ± 12.25	64.18 ± 11.85	61.37 ± 12.87	0.23
**WC (cm)**	107.37 ± 10.08	107.09 ± 9.26	105.58 ± 9.51	0.32
**WHR**	0.93 ± 0.07	0.94 ± 0.07	0.92 ± 0.08	0.06
**SBP (mmHg)**	125.05 ± 15.83	119.74 ± 14.37	122.98 ± 17.64	**0.04**
**DBP (mmHg)**	83.54 ± 10.96	79.64 ± 11.05	81.61 ± 12.75	**0.04**

*SES* Socio-economic status, *BMI* Body Mass Index, *FM* Fat Mass, *FFM* Fat free mass, *WC* Waist Circumference, *WHR* Waist-to-Hip ratio, *SBP* Systolic Blood Pressure, *DBP* Diastolic Blood Pressure; values are presented based on mead (SD). Comparison was done using chi-square and One-Way ANOVA. *P values based on ANCOVA adjusted for age and sex. Bold values provide the significant threshold of P < 0.05.

**Table 2:** Comparison of biochemical values in AGEs tertiles.

Variables	AGEs (kilo unit/gram)			P*
	Tertile 1 (< 2.45)	Tertile 2 (2.46–3.58)	Tertile 3 (> 3.59)	
TC (mg/dL)	194.42 ± 38.32	191.67 ± 35.61	189.33 ± 36.90	0.59
HDL (mg/dL)	43.24 ± 9.93	43.65 ± 9.04	43.78 ± 9.71	0.90
LDL (mg/dL)	125.15 ± 34.00	124.59 ± 31.70	120.94 ± 30.70	0.57
TG (mg/dL)	42.00, 765.00	33.00, 417.00	38.00, 768.00	0.18
Glucose (mg/dL)	64.00, 193.00	50.00, 125.00	64.00, 314.00	0.24
Insulin (U/mL)	2.20, 81.5	1.5, 65.10	1.80, 150.80	0.49
HOMA-IR	0.49, 15.09	0.25, 18.49	0.29, 32.02	0.40
QUICKI	0.32 ± 0.03	0.33 ± 0.03	0.33 ± 0.03	0.67
Ag-RP (pg/mL)	29.44 ± 14.28	29.25 ± 17.28	34.15 ± 22.87	0.25
α-MSH (ng/L)	199.68 ± 137.01	202.09 ± 150.41	249.76 ± 200.35	0.17

*TC* Total cholesterol, *HDL* High density lipoprotein, *LDL* Low density lipoprotein, *TG* Triglyceride, *Ag-RP* Agouti related peptide, *α-MSH* α-Melanocyte stimulating hormone. Values are presented based on mead (SD) or median (min, max). Comparison was done using One-Way ANOVA and Kruskal–Wallis. *P values based on ANCOVA adjusted for age and sex.

**Table 3:** Comparison of daily macronutrients intake/ calorie intake across AGEs tertiles.

Variables	AGEs (kilo unit/gram)			P*
	Tertile 1	Tertile 2	Tertile 3	
CHO/Cal.%	60.26 ± 7.74	59.12 ± 6.14	54.85 ± 6.90	< 0.001
Pro/Cal%	12.22 ± 2.39	13.20 ± 1.68	13.43 ± 1.74	0.005
Fat/Cal%	29.96 ± 7.94	30.26 ± 6.23	34.56 ± 5.89	< 0.001
Total fiber (gram)	22.36, 311.76	16.21, 195.92	9.86, 200.64	< 0.001
Meats group (gram)	0.13, 5.81	0.59, 9.92	0.48, 7.90	0.006

*CHO* Carbohydrate, *Pro* Protein. Comparison was done using One-Way ANOVA and Kruskal–Wallis. *P values based on ANCOVA adjusted for age and sex.

**Table 4:** Comparison of dietary AGEs content in different FADS2 genotypes.

Variables	Genotype			P*
	CC	CT	TT	
Dietary AGEs content (kilo unit/gram)	38.38 ± 7.39	37.23 ± 7.15	39.43 ± 6.62	0.43

*P values based on One-Way ANOVA.

## Discussion

To the best of our knowledge, it is the first study that investigated the modifying role of dietary AGEs content in different FADS_2_ genotypes in terms of obesity-related risk factors. Failure of success in one-dimensional interventions for obesity, like energy restriction or increased physical activity level to enhance energy expenditure, might be an appropriate reason to conduct such studies that evaluate the simultaneous effects of dietary ingredients and genetic susceptibility and to design personal dietary interventions according to genetic makeup. Based on our findings, older adults had lower amounts of dietary AGEs intake; it is quite clear that older adults are more cautious and sensitive to their food choices. It may be attributed to the fear of experiencing chronic disease such as diabetes mellitus, cardiovascular disease and hypertension in the future or having more time to devote for cooking and not using processed foods which affects food preferences to choose low fat and low sugar foods. Also, younger adults prefer more grilled or fried foods instead of boiled ones. These findings are in line with results of Ghorbaninejhad's et al. study conducted in Iranian population^35^. In addition, the socio-economic status of society acts like a double-edged sword. Indeed, people with low SES consume more dietary carbohydrate sources such as bread particularly high amounts of CML, the most known and prominent AGEs. In contrast, people with higher SES have higher consumption of processed foods and meat products that are high in fat and protein. Subsequently, cooking meat and poultry with high amount of lipid and protein in dry heat, facilitates the production of reactive protein and lipid compounds with covalent bond between reduced sugar and free amino group of protein, nucleic acid and lipids that results in production of high amount of AGEs^12^. In overall, mean dietary AGEs intake among study participants was estimated as 9522 (KU) daily, while it is significantly lower than other populations with higher adherence to western diets^36^, which may be related to different methods of preparation and preservation of foods. Similarly, prior studies that were conducted in Iran have reported lower dietary intake of AGEs^35,37^. In addition, although there are some cultural differences between Iranian and American food habits, food items with high amount of AGEs including meat, meat substitute, poultry, fish, nuts and fats group are available in both of Iranian FFQ and the American food’ AGEs database; we also considered different preferred cooking methods of Iranians in choosing food items from the table. Only some minor differences were found in vegetables and fruits, that because of their low AGEs content, it doesn’t seem to affect the results dramatically. Although, food habits are one of the most complex aspects of human behavior and are controlled by numerous physiological and environmental factors, but, even in diverse geographical distribution of population, there are similar perspectives on healthy eating definition. Likewise, excessive consumption of AGEs and their accumulation in human tissues induces RAGE to evoke oxidative stress and inflammation associated with obesity, atherosclerosis, diabetes and cardiovascular diseases^38,39^. Meanwhile, no significant difference was reported in terms of biochemical and anthropometric values except for FM, SBP and DBP in AGEs tertiles; obese adults tended to show lower level of FM, SBP and DBP in moderate intake of AGEs. In another glance, reduced carbohydrate intake is evident in the highest tertile of AGEs among obese adults. Uribarri et al. suggests that it may be related to higher water content or higher level of antioxidants and vitamins in carbohydrate-rich foods^16^. In addition, Vistoli et al. had stated that water activity, pH and size of sugar reactants, time and temperature of cooking may alter AGEs content of foods^40^. It is clear that plant-based foods that are rich in carbohydrate such as legume, vegetable, fruits and grains, have low AGEs content, so, the role of carbohydrate nature and cooking methods cannot be ignored in this regard which is consistent with our results. Contrarily, there is an uptrend of protein and fat intake in the highest tertile of AGEs. This finding confirms that foods high in protein and fat often consist of animal sources with high amount of lysine and arginine residues, that together with tryptophan, cysteine and histidine, are considered as potential targets for glycation^41^ and contribute to increased risk of obesity^42,43^ and metabolic syndrome^44,45^. Similarly, developed adiposity and insulin resistance have been tracked with consuming AGEs rich foods in mice^46^. It is elucidated that, TT carriers had higher adherence to western diet with high dietary AGEs content in comparison to heterozygote genotype (CT). In moderate intake of AGEs, all genotypes could modify the adverse effect of AGEs on FM compared to lowest intake. However, in high consumption of AGEs-rich foods, the adverse effect of AGEs becomes more prevalent and dominant. In this sense, evidence indicate that increased intake of dietary AGEs leads to accumulation of AGEs and expression of RAGE in adipose tissue with high amount of macrophages; as a result, high amount s of pro-inflammatory markers will be produced and obesity is the final consequent. Thereby, dietary AGEs have been introduced as drivers of oxidative stress and activators of AGE-RAGE axis to initiate obesity-related inflammation^12^. Obesity is associated with increased chronic low-grade inflammatory status; while the production of several inflammatory parameters are triggered by increased amount of AGEs; Ag-RP and α-MSH, as markers of pro-inflammatory status, are highly affected by AGEs content of body and their increased production has been proved previously. As reported in our results, Ag-RP and α-MSH were increased in high tertiles of dietary AGEs; although this increase was statistically non-significant, but it can be clinically important finding. Although the contributing mechanism of the role of fatty acids in the Ag-RP and α-MSH gene expression and consequent AGEs production is not fully understood, however, previous studies demonstrated that saturated fatty acids which are sources of AGEs production, promote inflammatory status through activating TLR4 and its downstream inflammatory responses including IkB kinase (IKK) and Jun N-terminal kinases (JNK) signaling^47^. Likewise, all of these processes contribute to stimulation of Ag-RP expression and promote food intake. It has been elucidated that hypothalamic inflammation induced by fatty acids leads to obesity and it can induce insulin and leptin resistance which are involved in the regulation of food intake and obesity^21,47^. Interestingly, we saw an increasing trend in FM value in all genotypes in high tertiles of AGEs compared to moderate intake. With regard to gene-diet interactions, TT carriers were more prone to have higher WC despite consuming low dietary AGEs; they also had higher level of blood cholesterol by increased dietary AGEs intake. Despite the beneficial role of CT genotype, moderate intake of AGEs, has increased weight and WC compared to lower intakes of AGEs and this genotype was not capable to attenuate adverse effects of dietary AGEs on weight and WC; but in general, CT carriers tended to use lower amount of dietary AGEs compared to other genotypes. Independent of genetic susceptibility, Ghorbaninejad et al. reported reduced weight in high dietary intake of AGEs which was in accordance with our findings^35^. To the best of our knowledge, the current study investigated the simultaneous effects of gene-diet interaction in terms of dietary AGE on obesity- related metabolic traits for the first time. Nevertheless, there is no documented dietary AGEs content database specified for Iranian population, which is considered as a potential limitation of the current study. Regarding to dietary differences, some food items which are consumed by Iranians, were not available and their AGEs contents were missed or replaced by most similar food items. On the other hand, the cross-sectional design of the study, had limited the understanding of causal relationships. In addition, using FFQ to estimate dietary intakes, might be a potential source of recall bias; however, we used a validated and reliable FFQ to minimize this possibility.

## Conclusion

The current study indicated that the total amount of dietary AGEs in Iranians might be lower compared with previous studies in western countries. Accordingly, higher consumption of protein or fat-based foods constitute high amount of AGEs and heterozygote genotype for FADS_2_ tended to show lower level of dietary AGEs content. Gene-diet interactions elucidated significant modifying effects on weight, FM, WC and cholesterol. These findings deserves further investigation in this concept to develop new approaches that are crucial for preventing metabolic abnormalities.

## Supplementary Information

Supplementary Information.
