# Supplementary material for: Personalized diet study of dietary advanced glycation end products (AGEs) and fatty acid desaturase 2 (FADS2) genotypes in obesity
Source: Sci Rep. 2021 Oct 5;11:19725. doi: 10.1038/s41598-021-99077-3 (PMC8492634; doi:10.1038/s41598-021-99077-3)
Supplement: Supplementary file 1 — Supplementary Information. [file 41598_2021_99077_MOESM1_ESM.docx]

**Title: Personalized diet study of dietary advanced glycation end products (AGEs) and fatty acid desaturase 2 (FADS_2_) genotypes in obesity**

**Authors: Mahsa Mahmoudinezhad ^1^, Mahdieh Abbasalizad Farhangi ^1*^, Houman Kahroba ^2^, Parvin Dehghan ^3*^**

^1^ Department of Community Nutrition, Faculty of Nutrition, Tabriz University of Medical Sciences, Tabriz, Iran

^2^ Molecular Medicine Research Center, Tabriz University of Medical Sciences, Tabriz, Iran

^3^ Department of Biochemistry and Nutrition, Faculty of Nutrition, Tabriz University of Medical Sciences, Tabriz, Iran

*First Corresponding author e-mail: [abbasalizad_m@yahoo.com](mailto:abbasalizad_m@yahoo.com); [abbasalizadm@tbzmed.ac.ir](mailto:abbasalizadm@tbzmed.ac.ir); Second corresponding author: [dehghan.nut@gmail.com](mailto:dehghan.nut@gmail.com); Postal code: 5165665931

Attar-Neishabouri Ave, Golgasht St, Tabriz, Iran. Phone: +04133357584

**Supplementary Material**

**Supplementary Table 1.** Comparison of biochemical values in AGEs tertiles in a sex stratified model.

|  | **Women** | | | | **Men** | | | |
| --- | --- | --- | --- | --- | --- | --- | --- | --- |
|  | **T1** | **T2** | **T3** | **P*** | **T1** | **T2** | **T3** | **P*** |
| **Age (y)** | 43.94 ± 8.82 | 41.07 ± 9.31 | 39.50 ± 9.63 | 0.05 | 41.14 ± 8.38 | 39.31 ± 8.34 | 38.75 ± 9.35 | 0.30 |
| **BMI (kg/m^2)^** | 34.47 ± 5.80 | 33.02 ± 5.31 | 34.22 ± 4.94 | 0.39 | 31.82 ± 4.61 | 31.91 ± 3.70 | 31.33 ± 4.30 | 0.69 |
| **FM** | 39.44 ± 8.57 | 36.11 ± 6.73 | 40.00 ± 8.91 | 0.15 | 32.60 ± 8.33 | 27.84 ± 5.48 | 28.51 ± 8.26 | 0.03 |
| **WC** | 105.07 ± 10.41 | 102.28 ± 9.34 | 103.09 ± 10.24 | 0.37 | 109.51 ± 9.35 | 109.82 ± 8.07 | 107.59 ± 8.44 | 0.28 |
| **SBP(mmHg)** | 121.46 ± 14.97 | 117.85 ± 15.64 | 121.24 ± 15.71 | 0.46 | 128.39 ± 16.01 | 120.81 ± 13.54 | 124.38 ± 19.06 | 0.03 |
| **DBP(mmHg)** | 83.23 ± 10.99 | 78.78 ± 11.80 | 80.44 ± 11.07 | 0.15 | 83.83 ± 11.03 | 80.13 ± 10.66 | 82.56 ± 13.98 | 0.19 |
| **Glucose (mg/dl)** | 93.05 ± 13.29 | 89.11 ± 11.95 | 93.22 ± 12.15 | 0.22 | 94.55 ± 21.57 | 90.52 ± 11.29 | 95.75 ± 33.39 | 0.39 |
| **LDL (mg/dl)** | 128.45 ± 37.79 | 123.57 ± 38.40 | 118.37 ± 31.41 | 0.37 | 122.15 ± 30.16 | 125.17 ± 27.46 | 123.02 ± 30.21 | 0.82 |
| **HDL (mg/dl)** | 45.54 ± 10.49 | 46.76 ± 9.57 | 48.38 ± 10.04 | 0.37 | 41.14 ± 8.98 | 41.89 ± 8.29 | 40.08 ± 7.71 | 0.45 |
| **Cholesterol (mg/dl)** | 197.27 ± 38.82 | 190.47 ± 41.99 | 186.84 ± 38.25 | 0.40 | 191.82 ± 38.02 | 192.35 ± 31.72 | 191.35 ± 35.96 | 0.98 |
| **TG, (mg/dl)** | 141.78 ± 80.98 | 119.09 ± 49.07 | 132.90 ± 83.48 | 0.34 | 184.82 ± 125.64 | 146.28 ± 77.89 | 169.20 ± 106.37 | 0.10 |
| **Insulin** (U/mL) | 18.73 ± 10.01 | 15.40 ± 11.31 | 16.10 ± 11.68 | 0.41 | 14.68 ± 13.17 | 14.89 ± 8.42 | 16.59 ± 22.01 | 0.81 |
| **HOMA-IR** | 4.32 ± 2.32 | 3.55 ± 3.15 | 3.83 ± 3.11 | 0.53 | 3.41 ± 2.70 | 3.37 ± 2.01 | 3.99 ± 5.16 | 0.64 |
| **QUICKI** | 0.32 ± 0.03 | 0.33 ± 0.03 | 0.33 ± 0.03 | 0.37 | 0.33 ± 0.03 | 0.33 ± 0.03 | 0.33 ± 0.04 | 0.95 |
| Ag-RP (pg/ml) | 28.13 ± 14.28 | 23.75 ± 9.07 | 30.25 ± 21.65 | 0.30 | 31.35 ± 14.43 | 32.54 ± 20.09 | 39.36 ± 23.80 | 0.29 |
| α-MSH (ng/L) | 211. ± 150.21 | 163.44 ± 96.10 | 228.66 ± 193.54 | 0.25 | 182.62 ± 116.85 | 225.28 ± 171.99 | 277.90 ± 209.41 | 0.17 |

Data are presented as mean (SD).*Analysis of variance for continuous variables and χ^2^ test for categorical variables. Abbreviations: BMI, body mass index; WC, waist circumference; SBP, systolic blood pressure; DBP, diastolic blood pressure; LDL-C, low density lipoprotein cholesterol; HDL, high-density lipoprotein; TG, triglyceride; HOMA-IR, homeostasis model assessment of insulin resistance; QUICKI, quantitative insulin sensitivity check index; AgRP, agouti-related protein; α-MSH, alpha melanocyte stimulating hormone.


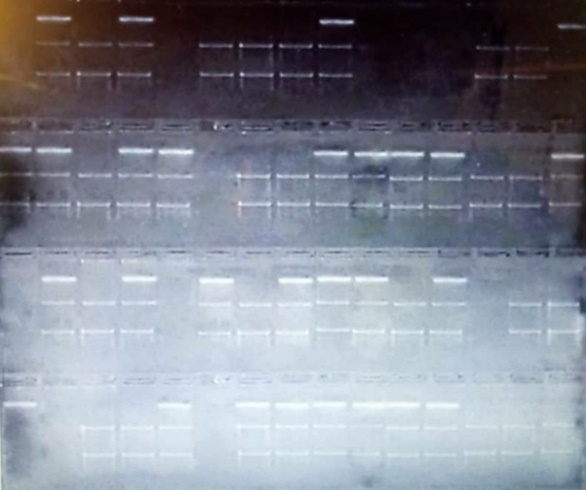

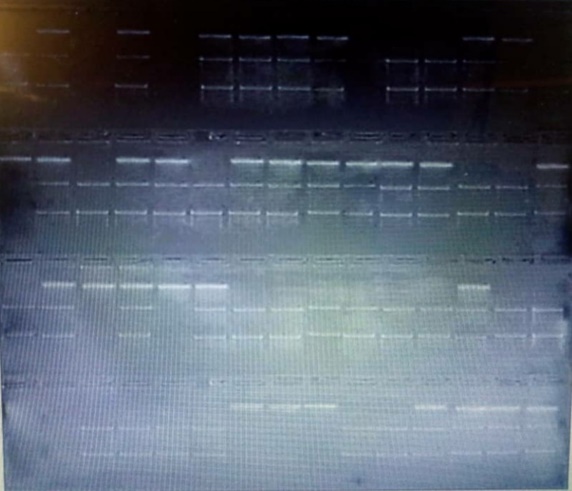

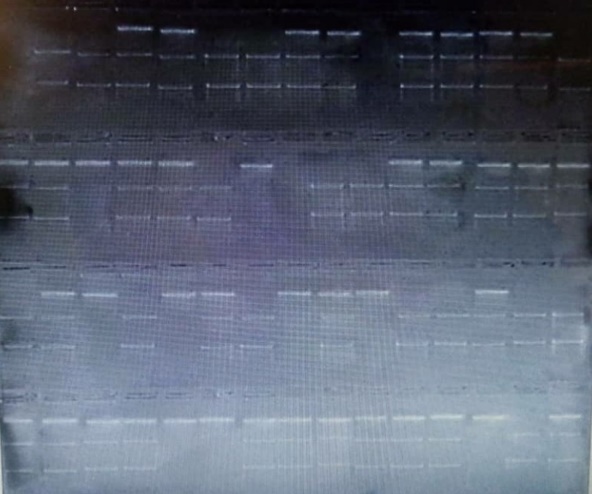

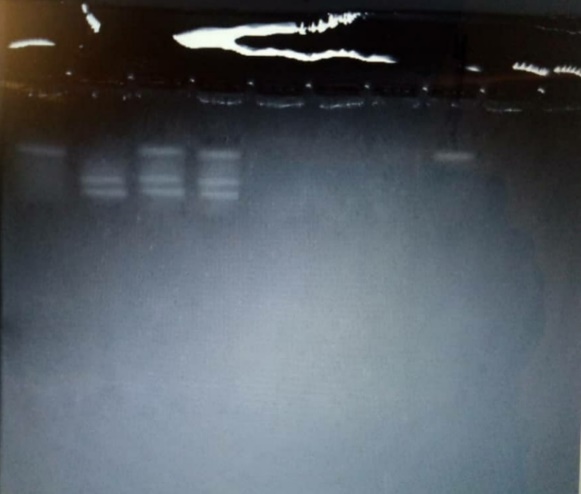


**Supplementary Figure 1. PCR analysis of multiple exposures for** rs174583 polymorphism
